# Capillary Wave-Assisted Colloidal Assembly

**DOI:** 10.1021/acs.langmuir.4c02794

**Published:** 2025-01-29

**Authors:** MaCayla J. Caso, Luis D. B. Manuel, Cameron Bachar, Minerva G. Schafer, Nicholas S. Lombardo, Gloria E. Alvarado, Alona Komarenko, Kiana Manoo, Ali Mehrnezhad, Kidong Park, Kevin M. McPeak

**Affiliations:** † Cain Department of Chemical Engineering, Louisiana State University, Baton Rouge, Louisiana 70803, United States; ‡ Department of Engineering and Industrial Professions, University of North Alabama, Florence, Alabama 35632, United States; § Division of Electrical and Computer Engineering, Louisiana State University, Baton Rouge, Louisiana 70803, United States

## Abstract

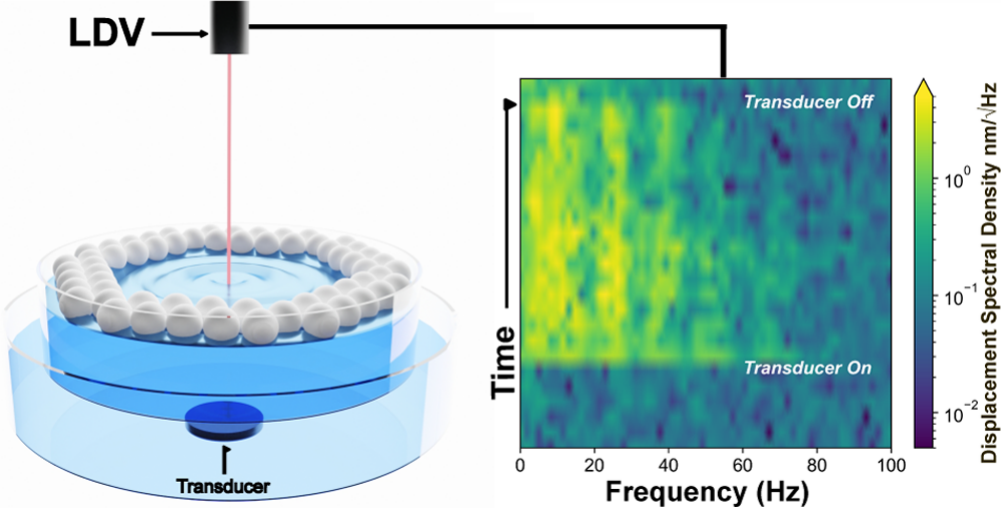

The self-assembly of nanoparticle colloids into large-area monolayers with long-range order is a grand challenge in nanotechnology. Using acoustic energy, i.e., acoustic annealing, to improve the crystal quality of self-assembled colloidal monolayers is a new solution to this challenge, but the characterization of the capillary waves driving the annealing process is lacking. We use a laser Doppler vibrometer and optical diffraction to uncover the frequency-dependent effects of capillary waves on the real-time self-assembly of submicrometer diameter polystyrene nanospheres at an air–water interface. Our study unambiguously demonstrates that low-frequency, e.g., sub-100 Hz, capillary waves are key to improving the long-range order of colloidal monolayers on an air–water interface. Furthermore, we demonstrate how a simple immersion transducer can generate capillary waves and how transducer placement and design affect vibrational spectra. Lastly, we show that frequency-shift keying of a high-frequency focused transducer provides a straightforward method of exciting low-frequency capillary waves that are effective at forming colloidal monolayers with excellent crystal quality, exhibited by grains over 3.5 cm^2^.

## Introduction

Colloidal assembly is a simple and rapid method for realizing colloidal monolayers over large surface areas, e.g., cm^2^. Controlling the long-range order of a colloidal monolayer on a surface has been studied for decades but still poses grand challenges. Overcoming these holds the promise of low-cost, rapid fabrication of ordered nanostructures that leverage collective phenomena, e.g., surface lattice resonances, with applications in biosensing, spectrometry, and photovoltaics.^1−8^ While multiple self-assembly methods exist to fabricate nanoparticle monolayers, i.e., dip coating, spin coating, and drop casting, self-assembly by the needle-tip flow method leverages the dispersion spread of a colloid on the water surface for rapid, large-area coverage.^9−12^ Colloids at an air–water interface have various forces acting on them that control their movement and ultimate assembly.^13^ Understanding how these forces interact is important for achieving large-area crystallinity.^14^ Van der Waals, electrostatic, hydrodynamic, and capillary forces act on the colloids and control the final nanoparticle arrangement.^13^ These forces cause attractive and repulsive interactions that lead to a thermodynamically favored minimum energy state.^13,15^ Importantly, self-assembly at an interface must accommodate both the thermodynamically favored state and the kinetics of the process. Kinetically driven defects can appear if there is not enough time for the colloids to arrange in a thermodynamically favored orientation.^16−18^

External stimuli, such as acoustic fields, electric fields, gas flow, and assisting self-propelled microparticles, allow the assembly to overcome kinetic trapping and thermal fluctuations caused by colloidal interactions.^19−22^ While acoustic fields have been shown to significantly increase the grain size of colloidal monolayers via acoustic annealing, open questions remain about the fundamental mechanisms driving this improvement. Recently, Shinotsuka et al. have shown that exposing colloidal monolayers to MHz sound waves, or low-frequency (sub-Hz) oscillations by barrier sway improved the crystallinity of the assembly up to 60 times, achieving grain sizes up to 7.52 cm^2^.^23,24^ Menath et al. investigated post growth acoustic annealing of colloidal monolayers by placing a beaker on top of a loudspeaker. Both Shinotsuka et al. and Menath et al. applied a range of frequencies and amplitudes to the colloidal assemblies to find the optimal conditions for large grain sizes.^23,24^ Shinotsuka et al. stated that the kinetic energy applied allows for the reorientation of the colloids but does not mention the role of acoustically driven capillary waves in the self-assembly process.^24^ Menath et al. concluded that circular surface waves with ∼1 cm wavelength provide interfacial space for the nanoparticles to reorient, but improvements in the grain size were highly dependent on the initial surface coverage of the colloidal monolayer.^23^ Based on these past findings, capillary waves play a critical role in acoustic annealing, but the characterization of these surface waves during acoustic annealing is lacking. Capillary waves exist on a fluid surface when excited by a vertically oriented vibration. Faraday first observed these waves in 1831 at an air-mercury interface and noticed their frequency was one-half the frequency of the vessel.^25^ More broadly, capillary waves are not restricted to the *f*/2 response observed by Faraday. This is especially true under high-frequency excitation sources, as used herein, where Faraday wave theory does not apply.^26^

In this article, we leverage operando techniques to directly investigate the effects of acoustic annealing on the real-time assembly of colloidal monolayers at an air–water interface. Using operando Laser-Doppler Vibrometer (LDV), we measure the frequency of capillary waves during acoustic annealing and draw connections between their frequency and displacement and the resulting improvement in the ordering of the colloidal polystyrene sphere (PS) monolayer. We show that low-frequency acoustically driven capillary waves are key to improving the ordering of self-assembled colloidal PS monolayers. Furthermore, we demonstrate the importance of acoustic annealing with a transducer operating in the far- vs the near-field regime. Transducers operating in the far field allow for more control over the frequency of the capillary wave and, ultimately, a better long-range order of the assembly.

## Methods

### Polystyrene Sphere Synthesis

We synthesized monodisperse PS with diameters of 550 and 800 nm following Reese et al.’s emulsifier-free emulsion polymerization method.^27^ We mixed 5.0 g of HEMA and 70 g of styrene in a water medium. 0.07 g of NaCl was added to synthesize the larger PS. We stirred the mixture at 350 rpm and kept it at 70 °C under a nitrogen blanket. We then added 0.11 g of potassium persulfate to 5 mL of water as an initiator for polymerization. The reaction was left to react for 24 h. After completion, we cooled the mixture to room temperature and then filtered. We centrifuged the suspension to a pellet, removed the supernatant, replaced it with pure water, and then sonicated it back into suspension. This cleaning was performed three times. We confirmed the diameter and coefficient of variance with a CPS Disc Centrifuge Model DC24000. Both sizes of PS had a coefficient of variance (CV) of less than 10%, see Figure S1 in the Supporting Information.

### Air–Water Interface Assembly

**Figure 1:**
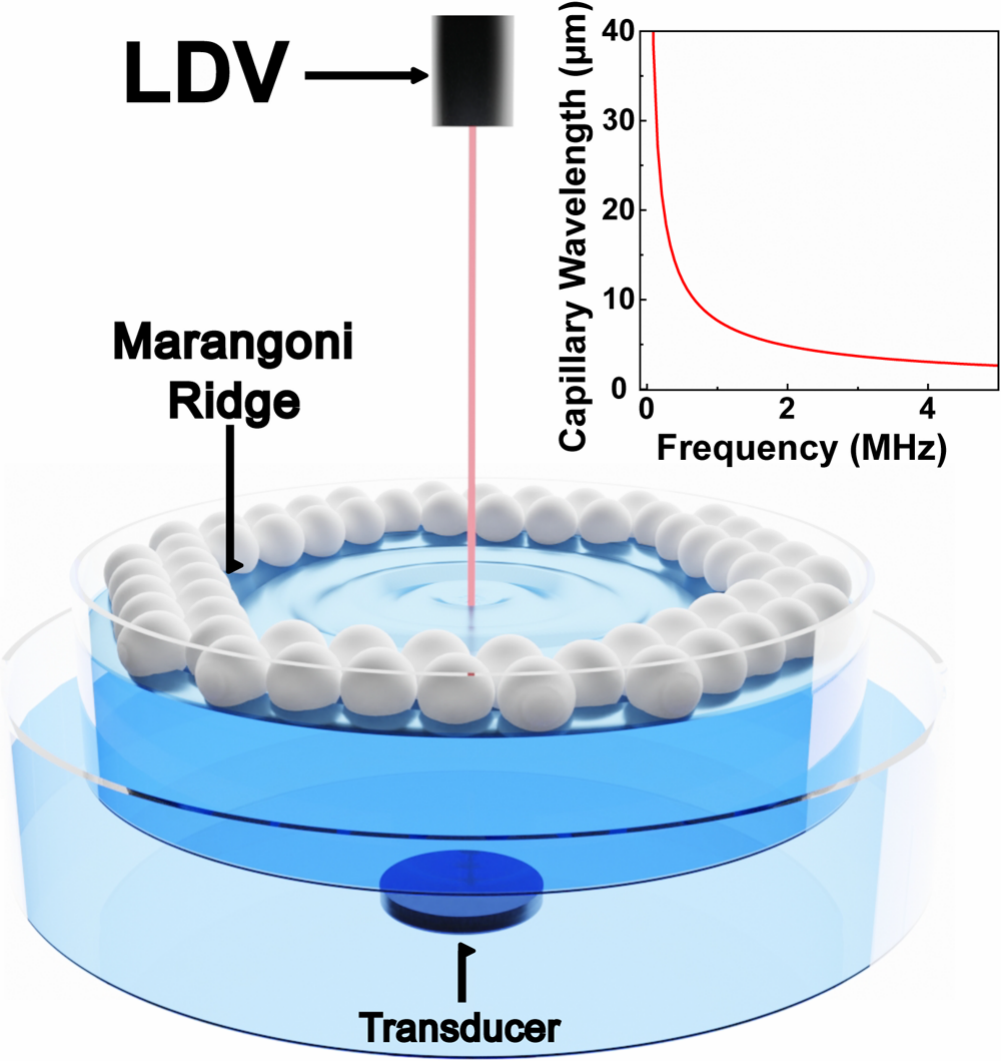
Capillary wave formation and characterization at the air–water interface. Schematic of the experimental setup for acoustically driven capillary waves and operando LDV measurements. The Marangoni ridge is labeled to show the edge of the PS as they pack. The inset is a plot of the capillary wave dispersion relationship, which allows us to calculate the wavelength of the capillary waves as a function of their frequency.

We used the needle-tip method introduced by Zhang et al. to deposit the PS onto the air–water interface (>280 cm^2^).^12^ We produced our assemblies in a 100 mm diameter polystyrene Petri dish filled with 18.2 MΩ deionized (DI) water, which was mounted partly submerged in a 140 mm diameter dish filled with tap water (Figures 1 and S2). We dispersed a 1 mL suspension of 50/50 by volume mixture of ethanol and PS (10 wt %) on the water surface through a 25-gauge needle at 13 μL/min. The needle-tip method allows for easier transference of the PS onto the air–water interface compared to other methods.^12^ We mounted a light-emitting diode (LED) light source next to the Petri dishes to highlight the structural color of the assembly.

For acoustic annealing, we placed the transducer in the center of the 140 mm dish beneath the smaller Petri dish that holds the assembly (Figures 1 and S2). Placing the transducer in a separate dish avoided contamination of the DI water used in the assembly with any residues on the transducer surface. Polystyrene has a low absorption coefficient, α = 100 × 10^–17^ s^2^/cm, at 2.5 MHz—the center frequency of the transducer—and presents minimal acoustic reflections due to reasonable impedance matching with water, i.e, a reflection coefficient (Γ) of 0.25, meaning 25% of the wave is reflected.^28,29^ We used a function generator (33220A, Agilent) connected to an RF amplifier (325LA, ENI) to drive the transducer, which provided 50 dB of gain. We set the function generator to a 150 mV_PP_ sine wave at 2.5 MHz unless otherwise stated. We used focused and flat broadband ultrasonic immersion transducers (IX-1457 and IX-1456, UTX, Inc.) with PZT4 material, 2.5 MHz center frequencies, 303 stainless steel housings, 17.78 mm diameters, and depths of 12.7 mm. The focused transducer had a focal length of 7 mm. We used frequency-shift keying (FSK) in the function generator to create tunable low-frequency capillary waves with the focused transducer. We varied the FSK rate from 5 to 200 Hz with the FSK hop frequency set to 100 Hz and the drive frequency fixed at 2.5 MHz. Setting the FSK hop frequency to 100 Hz, well outside the operating range of the transducer, pulsed the transducer at the FSK rate, enabling tunable low-frequency capillary waves.

### Air–Water Interface Detection

The position of the needle tip with respect to the air–water interface is critical for achieving a well-packed monolayer.^12^ We designed a device for repeatable needle placement at the air–water interface, as seen in Figure S3. The device holds a needle 1 mm above the focal spot of an S-polarized laser incident on the water surface at 60°. A photodetector is positioned at −60° to the water in the plane of incidence. We determined the *z*-position of the air–water interface by moving the stage in the *z*-direction until the maximum photodetector signal was achieved. We then lowered the needle to the air–water interface.

### Characterization of Polystyrene Assembly

We performed operando characterization of the PS assembly at the air–water interface by optical diffraction in transmission. We placed a mirror under the Petri dishes at a 45° angle with respect to the water surface. We mounted a laser parallel to the water surface, allowing for diffraction in transmission. We chose lasers with wavelengths of 405 nm (blue) and 532 nm (green) for 550 and 800 nm PS, respectively. Since the PS monolayer functions as a diffraction grating and we are measuring at normal incidence, the wavelength of the laser needs to be less than the pitch of the grating to observe diffraction orders greater than or equal to one. The pitch of the grating is equivalent to the diameter of the PS.

### Laser-Doppler Vibrometer (LDV)

LDV is a noncontact method of measuring vibrations on a surface, e.g., capillary waves, by detecting the Doppler shift^26^ from a laser reflected off the surface, as seen in Figure 1.^24^ We used two Polytec LDVs for the measurements: 1) OFV-5000 controller, VD-09 velocity decoder, with OFV-534 sensor head and 2) VibroFlex Connect VFX-F-110 front-end with VFX-I-130 sensor head and a VIB-A-510 illumination module with 5× and 20× Mitutoyo objectives and 633 nm HeNe laser to quantify the frequency and amplitude of capillary waves at the water interface.^23−25^ A 15 Hz high-pass filter was used for the low-frequency measurements in Figure 5. The depth of focus for the 5× and 20× objectives is 180 and 12 μm respectively, significantly larger than the displacement of the measured capillary waves, which is critical for accurate LDV measurements.^30^ Furthermore, our measured vibrations are solely from the capillary waves, not the transducer or the Petri dish, since both were positioned well outside the depth of focus of the objective.

## Results and Discussion

**Figure 2:**
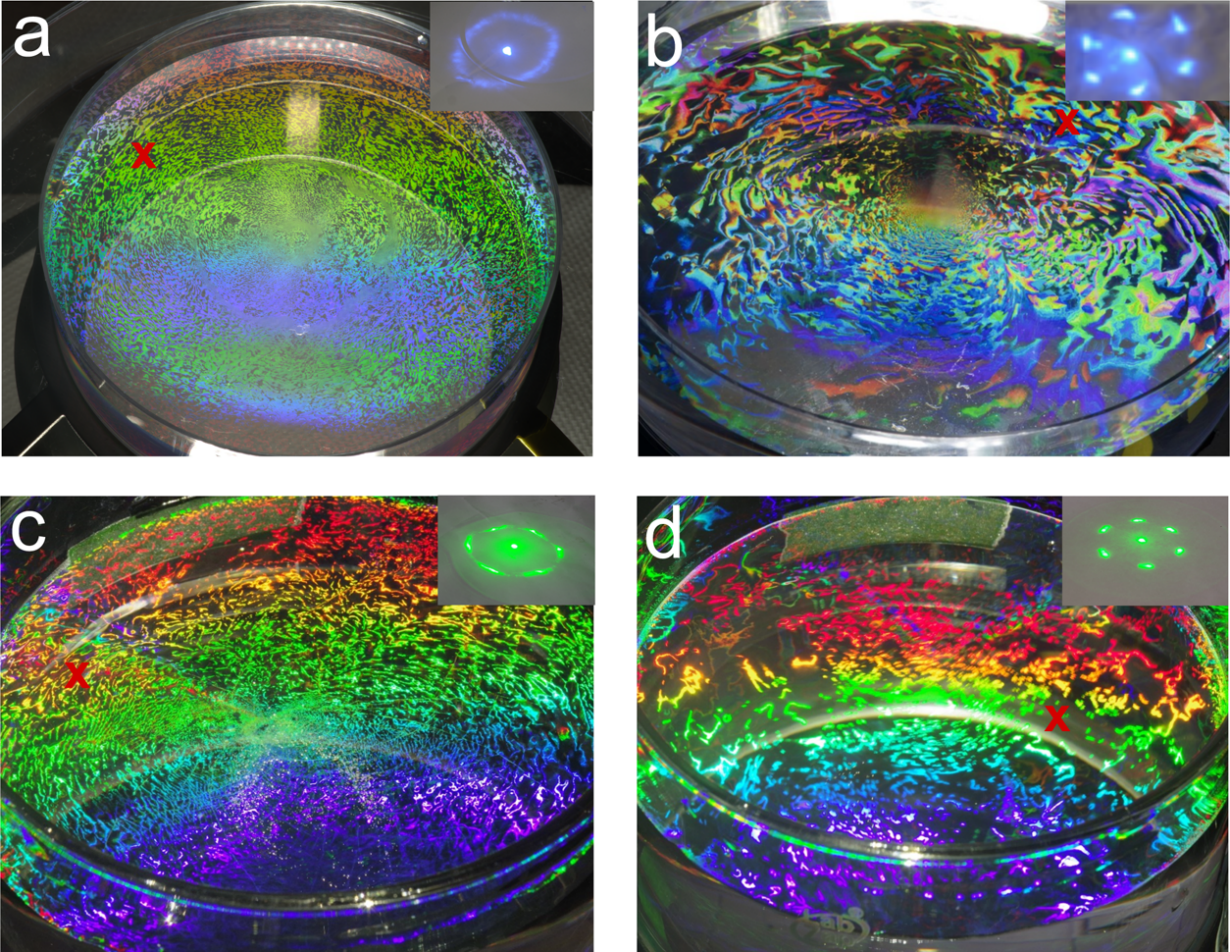
Flat transducer effects on PS assembly via the air–water interface method. Self-assembly of 550 nm PS spheres at an air–water interface using the needle flow technique (a) without capillary waves and (b) with capillary wave-assisted assembly using a flat immersion transducer operating at 2.5 MHz. Self-assembly of 800 nm PS spheres (c) without capillary waves and (d) with capillary wave-assisted assembly using a flat transducer operating at 2.5 MHz. The insets display the optical diffraction pattern generated at the red X for each assembly using 405 nm (a, b) and 532 nm (c, d) lasers.

We assembled control samples of 500 and 800 nm (Figure 2a,c) diameter PS at an air–water interface using a needle flow technique without the presence of capillary waves.^27^ The grains of the PS assembly are very small, as indicated by the color variation due to the differing crystal orientations of the grains on the sample. We measured the optical diffraction in transmission from the PS monolayers to assess their crystallinity better. The diffraction pattern in the Figure 2a,c insets shows only rings, indicating that the control colloidal monolayers have poor crystallinity. The rings are caused by optical interference in the scattering from different crystal orientations of the small grains. Next, we employed a flat transducer driven at 2.5 MHz during the assembly and observed a marked improvement in the diffraction pattern from the PS monolayer (Figure 2b,d). When the crystallinity of the monolayer is high, the diffraction pattern will display a distinct hexagonal pattern of dots, as seen in Figure 2b,d insets.^24^ The pattern of dots represents the crystal orientation of the nanosphere monolayer, showing the ordering of the (0,1), (1,0), and (1,1) planes of the crystal.^31^ Surprisingly, the 2.5 MHz flat transducer generated capillary waves that were easily observable by the eye.

To better understand the frequency–wavelength relationship of the observed capillary waves, we introduce the dispersion relationship for capillary waves (eq 1) and the wavelength–wavenumber relation (eq 2):
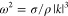
1
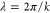
2

where ω is the angular frequency, σ is the surface tension of the air–water interface, ρ is the density of water, *k* is the wavenumber of the capillary wave, and λ is the wavelength of the capillary wave. We excluded gravitational effects in our dispersion model because we are primarily exploring capillary wave frequencies above 10 Hz.^26^ In the inset of Figure 1, we use eqs 1 and 2 to plot the relationship between the transducer frequency and the capillary wavelength, assuming synchronous coupling^12^ and an infinitely flat interface. For 2.5 MHz excitation, the capillary wavelength is expected to be less than 10 μm, which does not agree with our visual observation of the capillary waves.

**Figure 3:**
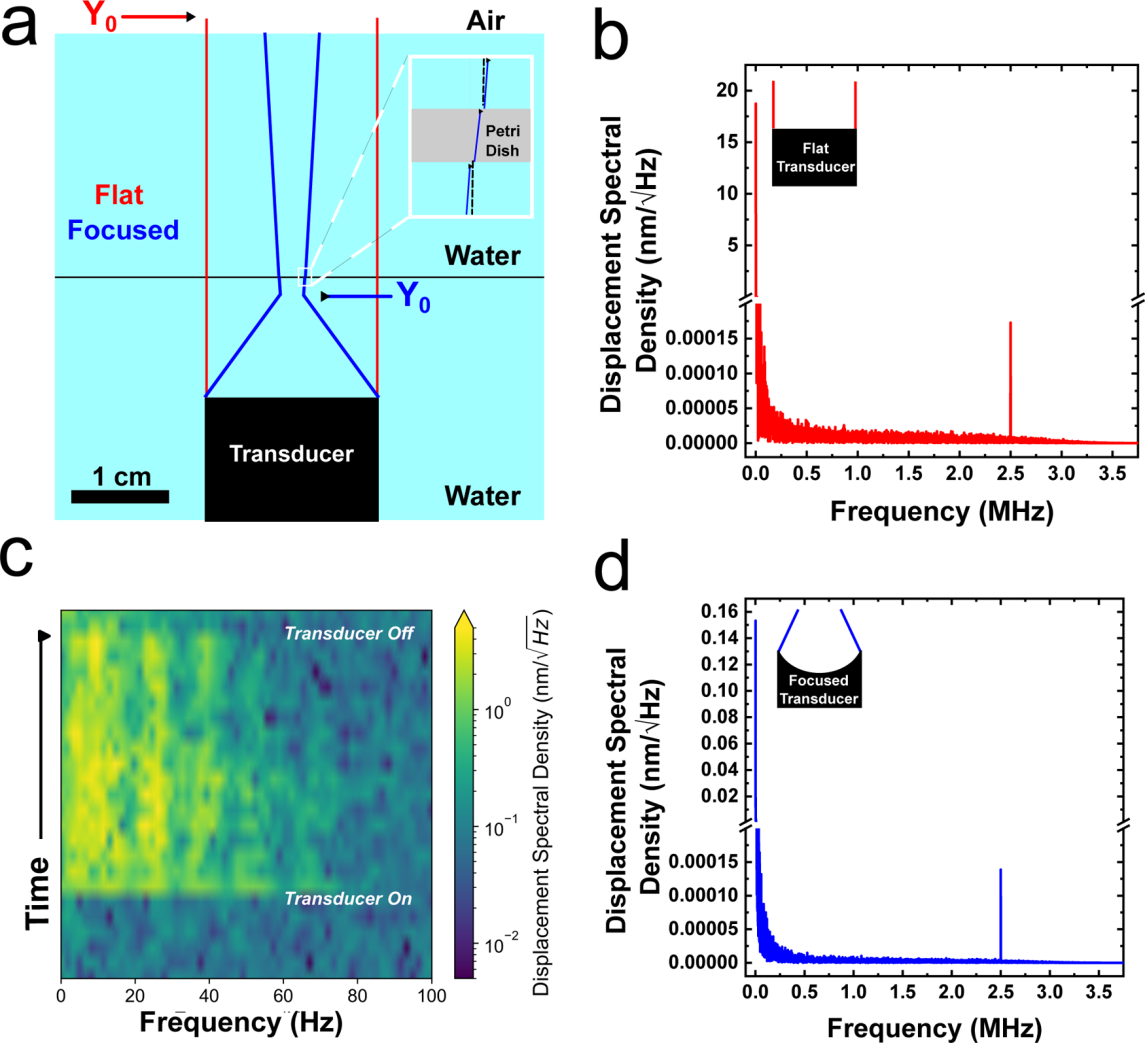
Transducer beam profiles and LDV measurements. (a) Beam profiles for the flat transducer (red) and the focused transducer (blue), *Y*_0_, mark the transition from the near to far-field region. The inset shows a slight shift in the direction of the acoustic wavefront due to the Petri dish. (b) Vibrational spectrum of capillary waves excited by the 2.5 MHz flat transducer. (c) Time-dependent vibrational spectrum of low-frequency capillary waves while power cycling the flat 2.5 MHz transducer. (d) Vibrational spectrum of capillary waves excited by 2.5 MHz focused transducer.

To understand why low-frequency capillary waves were observed, the beam spread of the transducer was investigated. Acoustic waves from a transducer go through two regions as they propagate through media: the near-field Fresnel region and the far-field Fraunhofer region. In the near-field region, there are irregularities, in the acoustic waves.^32^ The point where the near-field region becomes the far-field region is called the transition distance, *Y*_0*r*_, and is calculated with
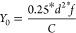
3where *d* is the transducer diameter, *f* is the frequency, and *C* is the speed of sound in the media. We calculated *Y*_0_ to be 6.83 cm for our flat transducer. In our flat transducer setup, *Y*_0_ is larger than the distance between the transducer and the water interface; thus, the acoustic waves remain in the near field during the assembly. In this near-field region, the acoustic waves propagate in a columnated space confined by the edge of the transducer, as seen in Figure 3a.^32^ Furthermore, in the near field, the acoustic wave experiences constructive and destructive interference, causing irregularities in the amplitude and frequency. We postulate that this leads to the low-frequency capillary wave irregularities we observed with the flat transducer. See Figure S4 for additional support for this hypothesis.

While we could visually see the low-frequency capillary waves, the 2.5 MHz excitation from the flat transducer should be synchronous with the interface, as predicted by Mahravan et al., resulting in both high- and low-frequency capillary waves.^33^ To verify this, we used LDV to measure the vibrational spectra of the capillary waves. Results of the LDV measurements from the flat transducer are shown in Figure 3b. We plot displacement spectral density (DSD) vs frequency to show the distribution of vibrational displacement energy across different frequencies. As predicted, we observed a peak for 2.5 MHz excitation from synchronous coupling and broadband low-frequency oscillations from capillary waves. To ensure that the low-frequency waves were formed from the transducer, we measured the time-dependent vibrational spectra of the capillary waves as we power-cycled the transducer. The LDV results show that when the transducer was turned on, the amplitude of a broad range of low-frequency capillary waves increased and decreased when the transducer was turned off (Figure 3c). This confirmed that the flat transducer exhibited high- and low-frequency capillary waves due to synchronous coupling with the 2.5 MHz acoustic wave and capillary wave turbulence from the near-field, respectively, as seen in Figure 3b.

We employed a focused transducer to understand whether the narrow-high- or broad-low-frequency capillary waves contributed to the enhancement in the crystallinity of the PS monolayer. A focused transducer concentrates the wave vectors as they propagate through the water,^32^ allowing only high-frequency capillary waves and a shorter *Y*_0_. *Y*_0_, calculated with eqs S1–S3, is below the surface of the water, allowing the air–water interface to be in the far-field region. The focused transducer beam profile is illustrated in Figure 3a. The air–water interface looked notably different between the focused and flat transducers. The focused transducer did not generate visible capillary waves, but a slight increase in optical scattering, which we posited was from micrometer-scale capillary waves. We confirmed this with LDV measurements that showed only 2.5 MHz capillary waves were present (Figure 3d). Note that the *y*-axis scale for the DSD in Figure 3d is 2 orders of magnitude smaller than that in Figure 3b.

**Figure 4:**
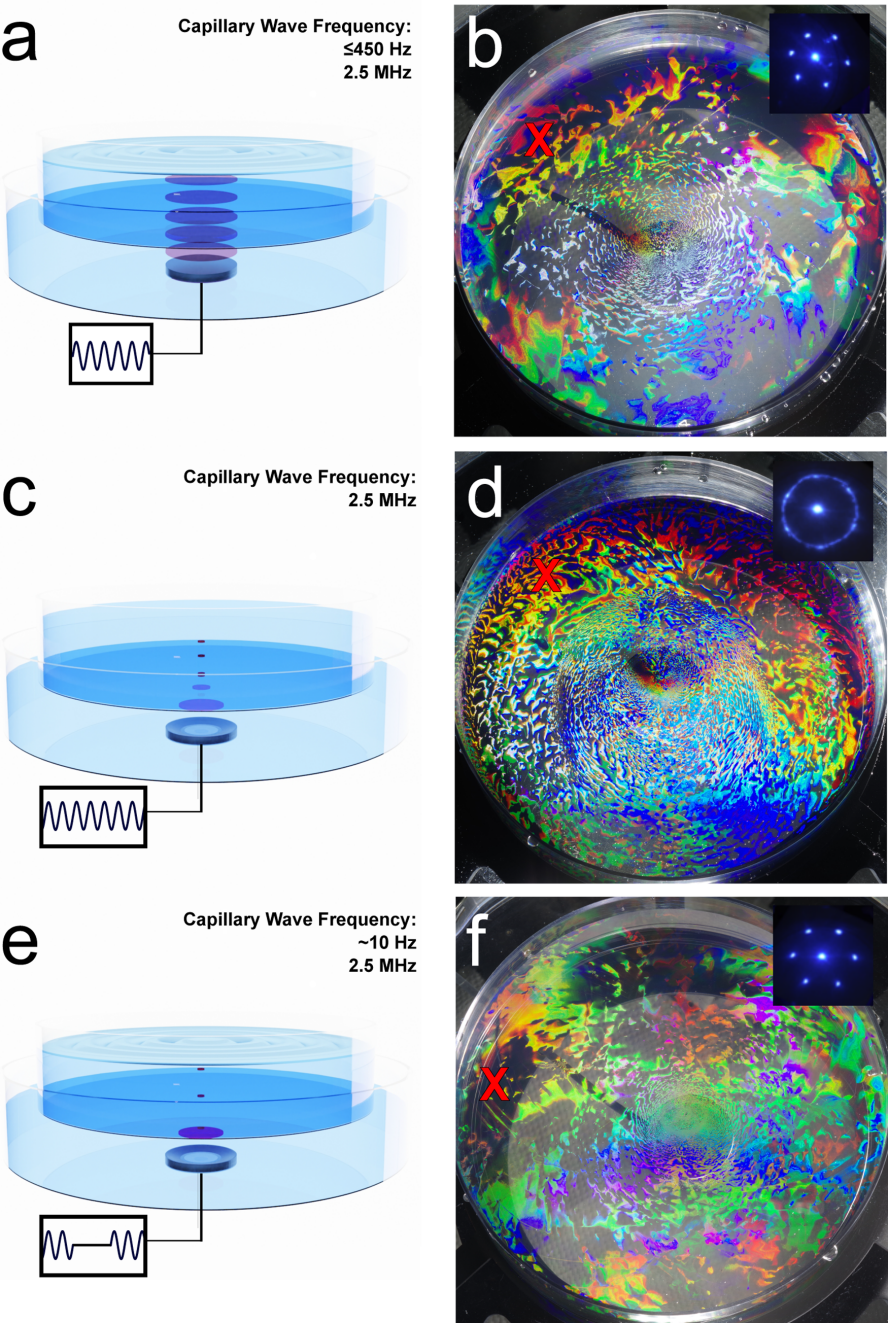
Acoustic annealing results from flat, focused, and pulsed-focused transducers. (a) Schematic of beam spread with a flat transducer. (b) Assembly of 550 nm PS spheres with a 2.5 MHz flat transducer. (c) Schematic of beam spread of a focused transducer. (d) Assembly of 550 nm PS spheres with a 2.5 MHz focused transducer. (e) Schematic of beam spread of a pulsed-focused transducer. (f) Assembly of 550 nm PS spheres with a 2.5 MHz pulsed-focused transducer. The insets in (b, d, f) display the optical diffraction pattern generated at the red X for each assembly using a 405 nm laser.

We compared the crystallinity of the monolayers from both low- and high-frequency-driven capillary waves to determine which resulted in more ordered PS monolayers. Figure 4 shows the different transducer arrangements that we tested, the capillary wave frequencies observed, and images of the resulting monolayers. The structural color in the images (Figure 4b,d,f) of the PS monolayers results from grating diffraction effects and indicates long-range order in the monolayer.^34^ Monolayers with larger grain sizes and fewer defects exhibit larger monochromatic regions. Therefore, we used the images as an initial assessment of the crystallinity of the PS monolayer. The focused transducer (Figure 4b) resulted in a significantly more disordered monolayer than the flat transducer (Figure 4a). This was further confirmed by the diffraction pattern (insets of Figure 4b,d,f) generated by a 405 nm laser beam in transmission. The hexagonally arranged dots in the inset of Figure 4b indicate long-range ordering, whereas the rings in the inset of Figure 4d are due to disorder in the monolayer. These results support the conclusion that low-frequency capillary waves are necessary for effective acoustic annealing. We then pulsed the focused transducer using FSK to excite narrow-band, low-frequency (sub-100 Hz) capillary waves. Large capillary wavelengths were observed on the interface, as predicted by the dispersion diagram in Figure 1. While the improvement in the crystal structure of the monolayers from low-frequency narrow-band vs broadband capillary waves was not as drastic as low- vs high-frequency waves, there was an improvement. The long-range order in the PS monolayer exposed to narrow-band low-frequency capillary waves resulted in a clear diffraction pattern, as seen in Figure 4f. We used ImageJ to calculate the pixel intensity and circularity (Figures S5 and S6) of the diffraction spots to determine the improvement in crystallinity of the monolayer.^35^ The use of diffraction is a standard method to quantify long-range order and defect density in crystals, where spot intensity and uniformity, or circularity, are dependent on the crystallinity of the material.^31^Figure S6a shows that the pulsed-focused transducer has the highest pixel intensity, with the lowest variation, showing a 20% increase in intensity from the flat transducer and over a 50% increase in intensity from the focused transducer. Figure S6b plots the circularity of the different assemblies. From the circularity metric, the monolayers produced by flat and pulsed-focused transducers are of equivalent quality, with the focused transducer generating far poorer monolayers. Considering both pixel intensity and circularity metrics, the pulsed-focused transducer produces PS monolayers with the highest crystallinity.

**Figure 5:**
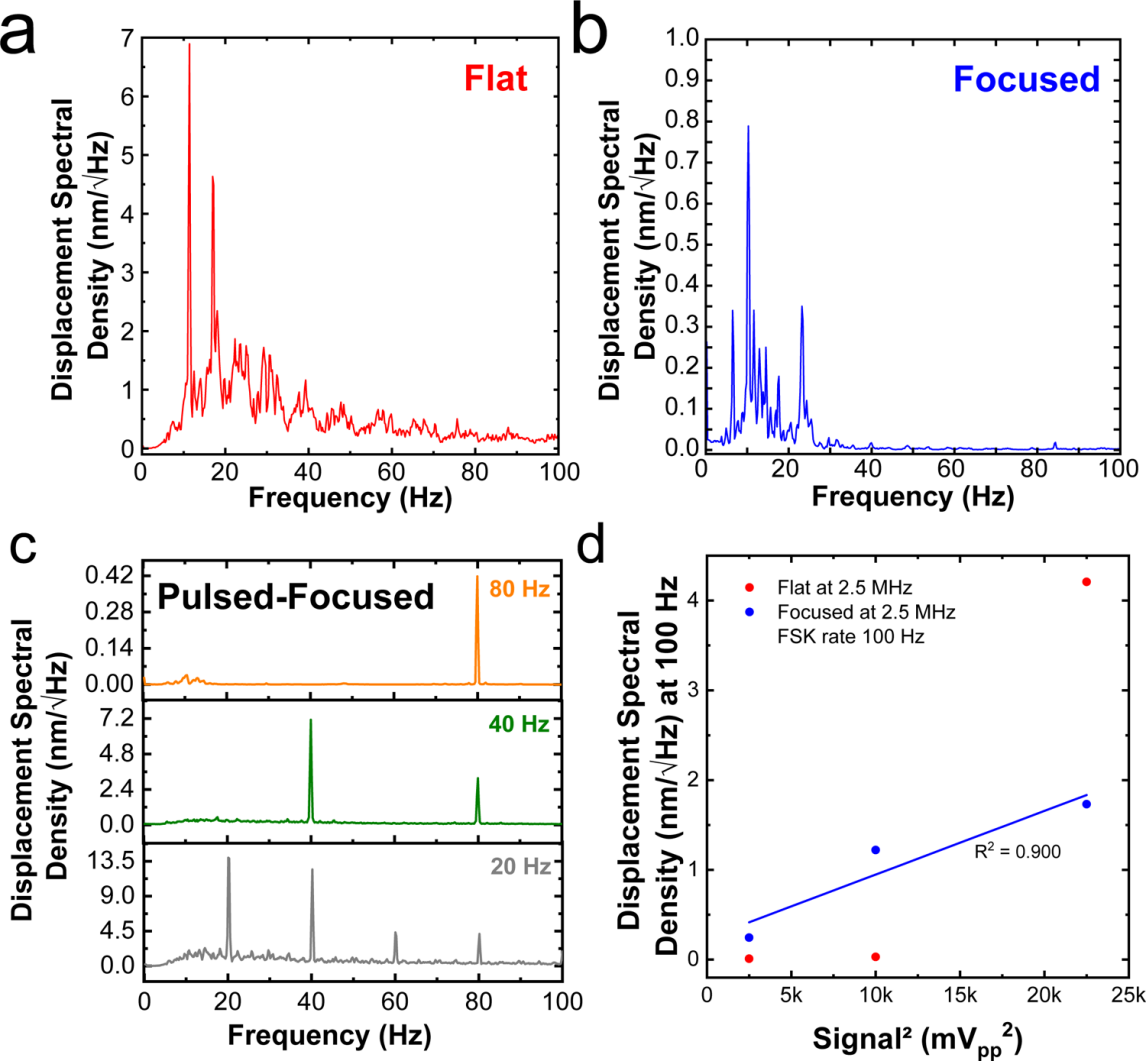
Vibrational spectra measured by LDV of low-frequency capillary waves excited at 2.5 MHz by the (a) flat transducer, (b) focused transducer, and (c) focused transducer at different FSK rates. (d) Displacement spectral density at 100 Hz for different powers delivered to the pulsed-focused transducer (FSK rate of 100 Hz) and flat transducer at 2.5 MHz.

To better understand the differences between the low-frequency capillary waves generated by the transducers, we plot their associated vibrational spectra in Figure 5. The flat transducer produces broadband waves spanning the low-frequency regime with a DSD of ∼5 mm/sqrt(Hz) at ∼18 Hz, corresponding to ∼20 nm of vertical displacement in the capillary wave (Figure 5a). As mentioned earlier by visual inspection, the focused transducer resulted in negligible low-frequency capillary waves (Figure 5b). Pulsing the focused transducer through FSK allowed us to precisely generate narrow-band capillary waves with broad tunability across the low-frequency regime (Figure 5c). Capillary waves driven with an FSK rate of 20 Hz had a DSD of 13.5 mm/sqrt(Hz) at 20 Hz, corresponding to a vertical displacement of over 60 nm. Capillary wave frequencies of 20 Hz achieved the best assemblies. Frequencies above 200 Hz reduced the grain size, often leading to the PS crashing into the subphase. Our results agree with the findings of Shinotsuka et al. and Menath et al., who both reported that low-frequency barrier sway or surface waves, respectively, led to the most significant improvement in the long-range order of the assembly.^23,24^ We posit that higher displacement values from the pulsed-focused vs the flat transducer resulted in improved crystallinity in the PS monolayers. Importantly, increasing the signal level to the flat transducer to boost the vertical displacement of the capillary wave increased the frequency of crashing the PS monolayer. To understand the mechanism for the capillary waves formed by the pulsed-focused transducer vs the nonpulsed flat transducer, we varied the signal voltage on the function generator (50, 100, and 150 mV_PP_). Figure S8 shows the corresponding vibrational spectra for the 2.5 MHz focused (pulsed with an FSK rate of 100 Hz) and flat transducers. Figure 5d shows that the DSD at 100 Hz for the pulsed-focused transducer is linearly proportional (*R*^2^ = 0.9) to the square of the excitation signal voltage, whereas the DSD for the flat transducer is highly nonlinear. Acoustic radiation force (ARF) is linearly proportional to the signal power, which is proportional to the square of the signal voltage.^36,37^ We posit that the ARF is excited at the FSK rate, pushing the water periodically and causing the narrow-band low-frequency capillary waves.

## Conclusions

We investigated the frequency-dependent effects of acoustically driven capillary waves on the real-time self-assembly of colloidal monolayers at an air–water interface. Operando LDV showed high, e.g., 2.5 MHz, and low, e.g., sub-200 Hz, frequency capillary waves from a flat transducer driven at 2.5 MHz. Synchronous acoustic to capillary coupling created the 2.5 MHz capillary wave, while broadband low-frequency capillary waves were generated by near-field effects. To confirm which frequencies were responsible for improving crystallinity, we employed a focused transducer to avoid near-field effects. Laser diffraction coupled with LDV measurements verified that low-frequency capillary waves, e.g., ∼20 Hz, were most effective at improving the long-range order of the monolayer. Pulsing the focused transducer with FSK enabled tunable narrow-band low-frequency capillary waves, which led to additional improvements in the monolayer ordering as compared to the broadband low-frequency waves from the flat transducer. We posit that the ARF drives the narrow-band capillary waves due to their linear relationship between DSD and the square of the signal. Acoustic annealing of colloidal self-assembly is a rapid method for achieving large-area, defect-free colloidal monolayers. Operando LDV is a powerful tool for measuring the vibrational spectra of capillary waves and correlating these spectra to improvements in the assembly process to guide further advances in the acoustic annealing of colloidal monolayers.

## Supporting Information Available

PS assembly (MOV)
Optical diffraction setup for assessing colloidal crystal quality; transducer placement; air–water interface detection; and supplemental figures (PDF)

## Supplementary Material

la4c02794_si_001.mov
