# Supplementary material for: Capillary Wave-Assisted Colloidal Assembly
Source: Langmuir. 2025 Jan 29;41(5):3033–41. doi: 10.1021/acs.langmuir.4c02794 (PMC11823604; doi:10.1021/acs.langmuir.4c02794)
Supplement: Supplementary file 2 — la4c02794_si_002.pdf [file la4c02794_si_002.pdf]

Supplemental Information:

Capillary Wave-Assisted Colloidal Lithography

MaCayla J. Caso,[1,2], Luis D. B. Manuel[1], Cameron Bachar[1], Minerva G. Schafer[1], Nicholas S. Lombardo[1], Gloria Alvarado[1], Alona Komarenko[1], Kiana Manoo[1], Ali Mehrnezhad[3], Kidong Park[3], Kevin M. McPeak[1]

[1] Cain Department of Chemical Engineering, Louisiana State University

[2] Department of Engineering and Industrial Professions, University of North Alabama

[3] Division of Electrical and Computer Engineering, Louisiana State University

Corresponding Author E-mail*: kmcpeak@lsu.edu

Contents:

Polystyrene Sphere Characterization 2
Setup for Acoustic Annealing 2
Air-Water Interface Detection 3
Transducer Placement 4
Beam Size and Focal Point Calculations 5
Laser Diffraction Setup for Assessing Colloidal Crystal Quality 6
Figures: 8
References: 9

Polystyrene Sphere Characterization

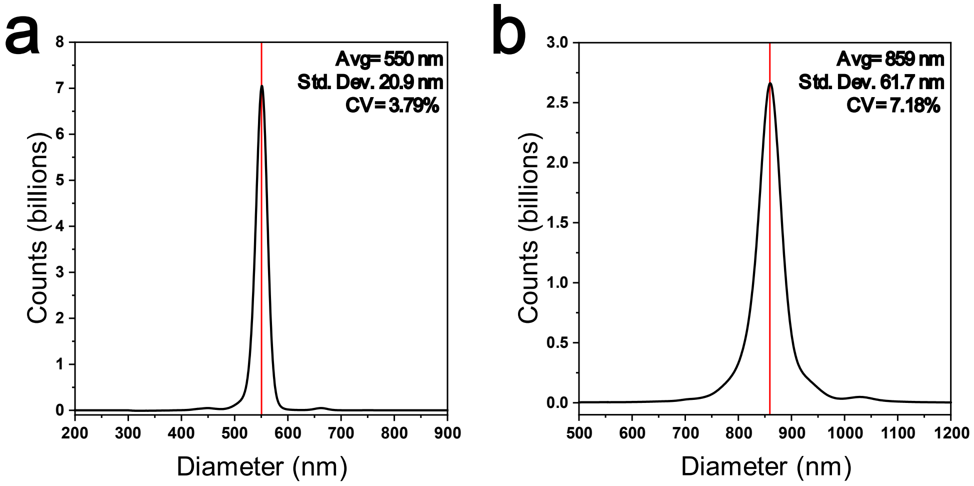

Figure S1. PS size distribution data. (a) 550 nm PS spheres diameter distribution. (b) 800 nm PS spheres diameter distribution

Setup for Acoustic Annealing

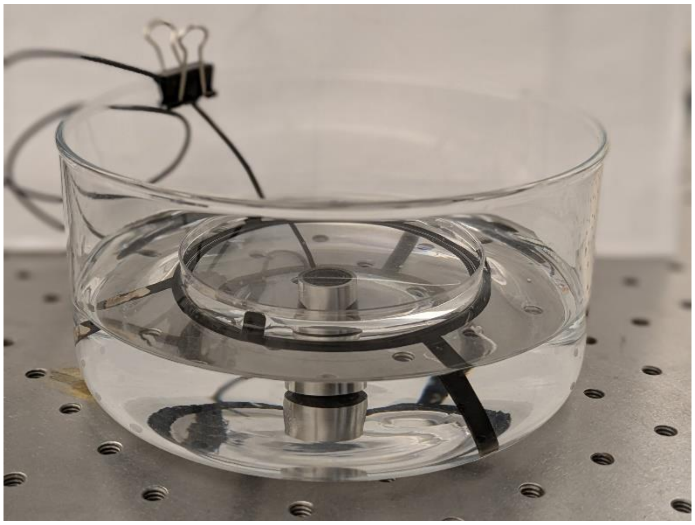

Figure S2. Photograph of the two-dish setup used for the acoustic annealing of the PS monolayers.

Air-Water Interface Detection

The z-height of the needle with respect to the air-water interface is critical for achieving a well-packed monolayer [S3]. The device for repeatable needle placement directly at the surface of the water, as seen in Figure S3, was first designed in AutoCAD. The goal of this device was to allow easy detection of the water's surface and have repeatable placement of the needle to form the meniscus needed for the needle-tip method to work properly [S3]. As shown in Figure S3, the device was designed to hold the needle in a fixed place along with a laser and a receptor connected to a spectrometer. The laser and receptor are held at fixed angles so that when the laser is at the air-water interface there is one spot that allows complete reflection of the laser to be detected by the spectrometer. This device was mounted to the original set-up made for the sonication assisted assembly method.

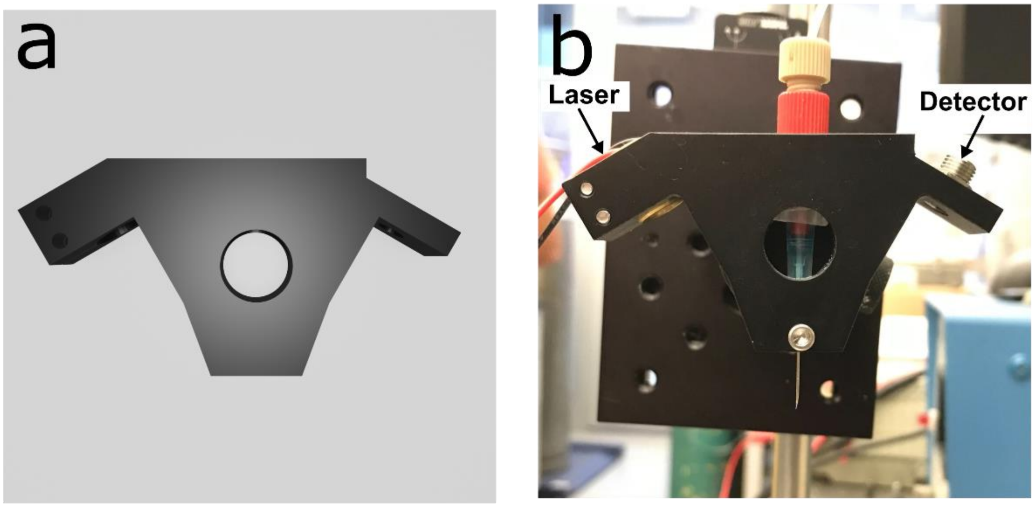

Figure S3. Needle-Tip Method. (a) AutoCAD needle-tip placement device. (b) Actual needle-tip placement device.

The water level was determined by adjusting the device location until a signal was seen on the spectrometer software. The device is then leveled and moved up and down until a peak signal is

displayed. Before a peak signal can be seen the device must be leveled to ensure the alignment of the laser to the receptor when at the water surface. Once the peak signal is seen the device can be lowered by 6.35 cm to place the needle at the water's surface.

Transducer Placement

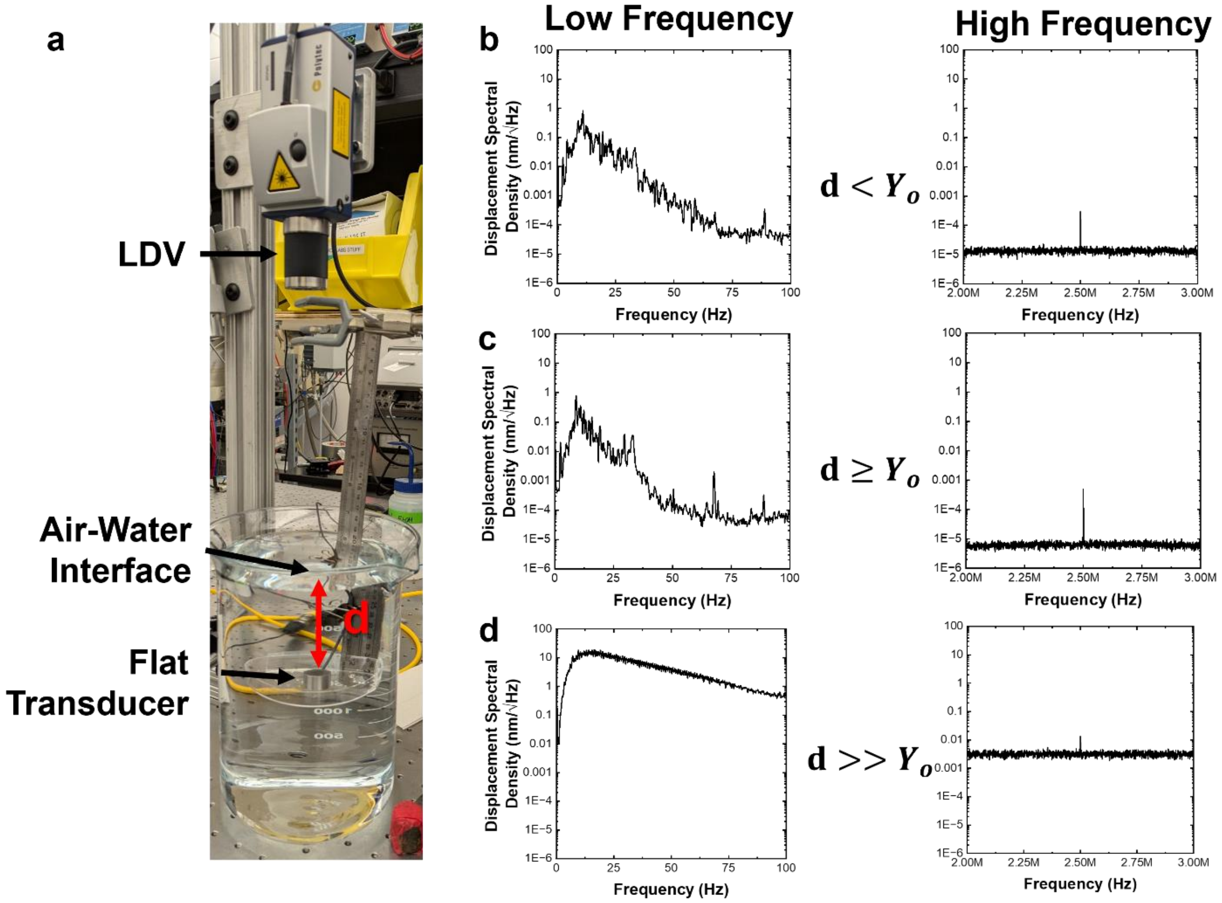

Figure S4. Setup and vibrational spectra for that flat transducer operating at 2.5 MHz at varying distances from the air-water interface, (a) photograph of the flat transducer with adjustable depth submerged in a large beaker with the LDV head mounted above. Vibrational spectra measured from the flat transducer mounted (b) within the near field ($d < Yo$), (c) in the transition region ($d \geq Yo$) and (d) in the far field ($d \gg Yo$). Note how the DSD in the low-frequency spectrum is greater at $d < Yo$ than for $d \geq Yo$. We are operating the flat transducer in the $d < Yo$ regime.

To investigate the effect the distance between our 2.5 MHz flat transducer and the air-water interface has on the capillary waves we immersed our flat transducer in a bucket of water and varied the distance between the transducer and the air-water interface. We confirmed that when our flat transducer is placed below the air-water interface at a distance (d) within the near field (i.e., $d < Y_0$), low-frequency capillary waves were visible. As we increased d beyond Y_0 (i.e., the far-field regime), low-frequency capillary waves were no longer visible, and the surface appeared similar to the 2.5 MHz focused transducer. If Y_0 was lowered even further, capillary waves were visible again due to the spread of the acoustic beam as it hit the surface.

Beam Size and Focal Point Calculations

The focal length of the focused transducer is 20 mm. The size of the focused beam (Eq. S1) and wavelength (Eq. S2) were calculated with:

$$d_f = 2.44 * \lambda * \frac{l_f}{d} \quad \text{(Eq. S1)}$$

$$\lambda = \frac{\left(\frac{d}{2}\right)^2}{z_r} \quad \text{(Eq. S2)}$$

Where l_f is the focal length and λ is the wavelength. For our focused transducer λ of the 2.5 MHz acoustic wave was calculated to be 0.61 mm and d_f was calculated to be 2.3 mm. Once at the focal length, the beam diverges at a set angle. This angle is calculated with:

$$\emptyset_d = \sin^{-1}\left(0.61 * \frac{2\lambda}{d}\right) \quad \text{(Eq. S3)}$$

Where φ_d is the angle of divergence. The φ_d for both flat and focused transducers were calculated to be 3.28˚. This angle begins in the far-field for the flat transducer, beyond the interface.

Laser Diffraction Setup for Assessing Colloidal Crystal Quality

A common method for determining the long-range order of a colloidal monolayer on an air-water interface is laser diffraction. We performed laser diffraction in transmission with 532 nm and 405 nm lasers. The laser light was parallel with the optical table and turned 90° upward using a small mirror mounted below the petri dish holding the colloidal assembly. The laser light passed through the colloidal assembly perpendicular to the air-water interface. We recorded the diffraction pattern on a screen mounted a few mm above the air-water interface. The diffraction pattern observed is the reciprocal lattice of the original PSs lattice structure, showing the (0,1), (1,0), and (1,1) diffraction orders of the crystal monolayer [S1]. With more order in the packing of the PSs the dots become more intense and pronounced [S2]. The spot orientation depends on the crystal's packing arrangement [S2]. For hexagonally packed assemblies, the diffraction pattern has six spots in a hexagonal pattern, corresponding to the (n,m) order diffraction spots around the center spot. This method is useful for real-time analysis of the ordering of assemblies at an interface. The analysis of this pattern is seen in Figure S5, which steps through the process of using ImageJ to find the pixel intensity of the dots. First, the images were adjusted to a threshold that only accounts for the area of the dots in the optical diffraction pattern. We then measured the pixel intensity over the area of the dots, which was given as a histogram of the data in Figure S5d. An average across the dots of the diffraction pattern was found and plotted for each transducer scenario (see Figure S6). Another measure of the ordering is the circularity of the dots. As greater order is present the dots become more circular, as seen in Figure 4b and 4f inserts, instead of looking more like rings, as seen in Figure 4d insert. This circularity can also be calculated using ImageJ. The circularity function provides a value from 0 to 1 as a ratio of the area and perimeter of the dot. As the value approaches one the dot is more circular . The same area used to quantify the pixel intensity was used for the circularity of the dots. The average circularity for the dots in each assembly was plotted in Figure S6.

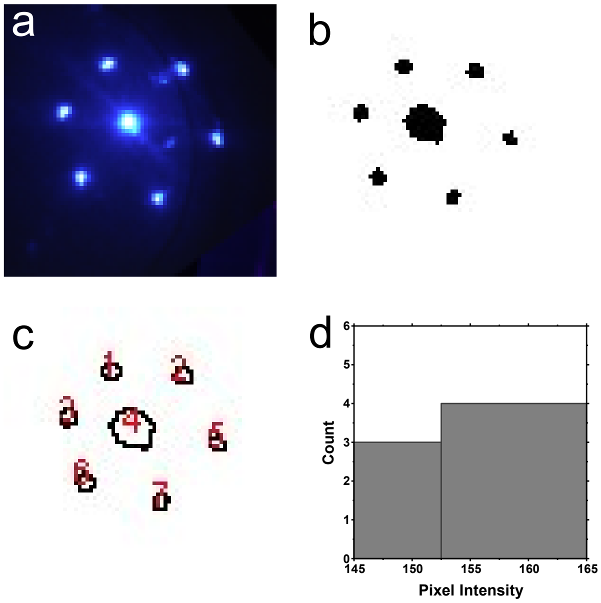

Figure S5. ImageJ analysis of laser diffraction patterns . (a) Real-time optical diffraction pattern from a PS monolayer. (b) Dot outline from threshold adjuster. (c) Overlay of dot analysis. (d) Pixel intensity distribution.

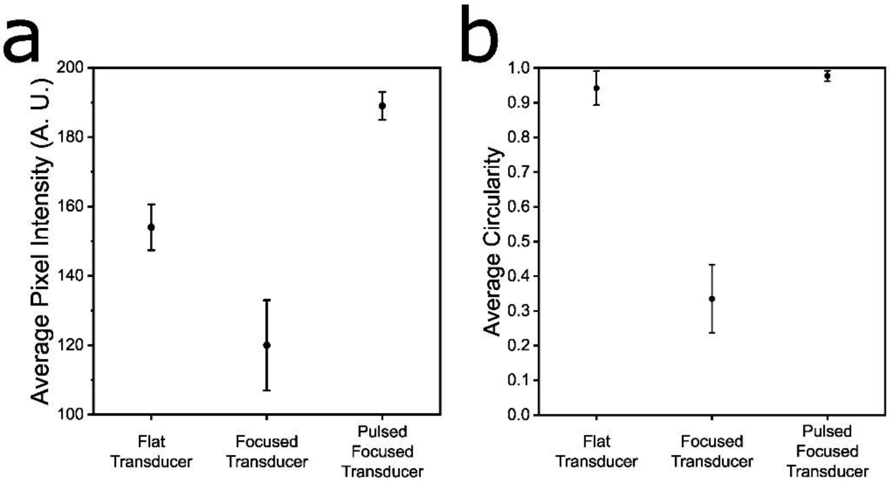

Figure S6. ImageJ analysis of laser diffraction patterns from the flat, focused, and pulsed focused transducer arrangements. (a) Mean pixel intensity of diffraction spots (b) Average circularity of diffraction spots

Figures:

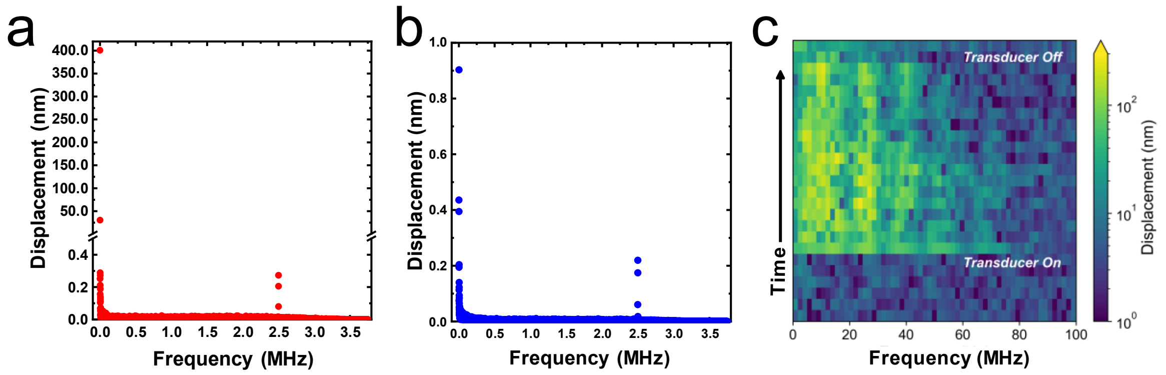

Figure S7. Capillary wave displacement vs. frequency measured by LDV for the (a) flat transducer at 2.5 MHz (b) focused transducer at 2.5 MHz. (c) Time-dependent plot of low-frequency capillary waves observed when the flat transducer is turned on and off.

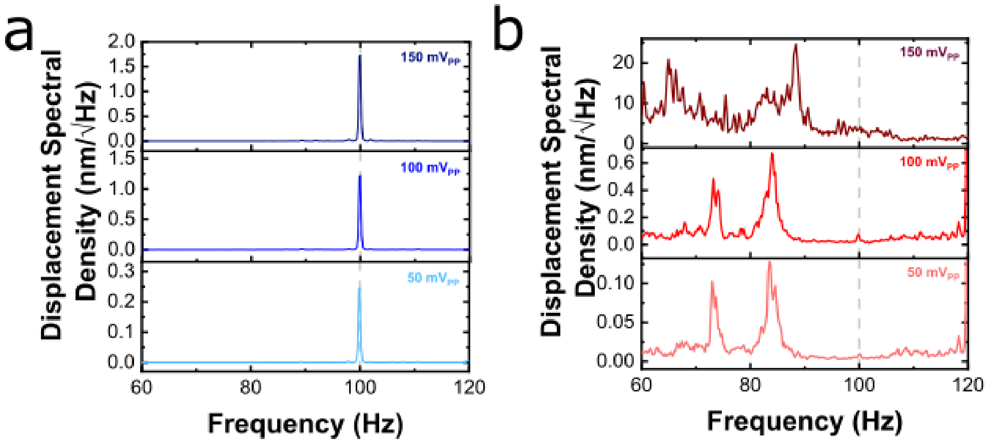

Figure S8. Effect of voltage signal amplitude on the LDV measured vibrational spectra for the (a) pulsed-focused transducer driven at 2.5 MHz with an FSK rate of 100 Hz and (b) the flat transducer driven at 2.5 MHz.

References:

S1. Whitesides, L. S. and G. M. *Structures of Self-Assembled Monolayer Films of Organosulfur Compounds Adsorbed on Gold Single Crystals: Electron Diffraction Studies*. Langmuir, 1988. **4** (3).

S2. Romanov, S.G., et al., *Engineered disorder and light propagation in a planar photonic glass*. Scientific Reports, 2016. **6**(1): p. 27264.

S3. Zhang, J.-T., et al., *Fabrication of Large-Area Two-Dimensional Colloidal Crystals*. Angewandte Chemie International Edition, 2012. **51**(25): p. 6117-6120.
